# Standardizing Measurement of Contraceptive Use Among Unmarried Women

**DOI:** 10.9745/GHSP-D-19-00298

**Published:** 2019-12-23

**Authors:** Madeleine Short Fabic, Apoorva Jadhav

**Affiliations:** a United States Agency for International Development, Washington, DC, USA.

## Abstract

Historically, the family planning practices and needs of married women have been monitored and reported uniformly. However, the same uniformity does not hold for unmarried women. Because key data and information platforms employ different measurement approaches—namely, different definitions of sexual recency—reports of contraceptive prevalence and unmet need among unmarried women are inconsistent. We examine how the measurement approaches employed by 3 large organizations yield such divergent estimates. We find that contraceptive prevalence and unmet need estimates among married women do not vary much by sexual recency. For unmarried women, contraceptive prevalence is systematically lower and unmet need is systematically higher as the sexual recency window widens. In the short term, we recommend using the 1-month cutoff as analyses reveal it yields the most precise estimates for better recognizing the needs of this important demographic group.

## Abstract (precis)

Because contraceptive prevalence and unmet need for family planning estimates for unmarried women vary widely depending on the chosen sexual recency inclusion factor, all data platforms should adopt a 1-month window in these calculations to have comparable and actionable estimates.

## BACKGROUND

With the world working to achieve the FP2020 goal that 120 million more women and girls access contraception and the 2030 Sustainable Development Goal that no one is left behind, understanding the family planning needs of all women is imperative.^1^ The contraceptive prevalence of married and in-union women has been monitored and reported in a relatively uniform way across time, place, and data collection instrument.^2^^,^^3^ The same cannot be said for the monitoring and reporting of family planning practices and needs among unmarried women.

When World Fertility Surveys began in the 1960s and 1970s, the approach to measuring contraceptive prevalence only among married and in-union women had merit due to cultural sensitivities of asking unmarried women about their sexual activity coupled with assumptions of low sexual activity among unmarried women.^4^ During the 1980s, this approach shifted, and it became standard to include unmarried women in population-based household surveys and other data collection platforms. This expansion was and remains important.

Although marriage is still largely the norm worldwide—according to the United Nations World Marriage Data, at least 80% of women aged 45−49 have ever married^5^—marriage is occurring later in life. Meanwhile, age at sexual debut remains relatively constant.^5^^,^^6^ Coupled with large youth populations in many low- and middle-income countries, this demographic shift yields not only a higher proportion of unmarried women and men exposed to the risk of pregnancy but also the highest number of unmarried individuals in human history.^7^ The increased focus on expanding family planning access among youth,^8^ who include a significant share of unmarried individuals, is clearly warranted. And the inclusion of unmarried women and men in key data collection platforms is imperative for understanding the family planning knowledge, attitudes, and practices among this growing demographic.

The key family planning data collection platforms—Demographic and Health Surveys (DHS), Multiple Indicator Cluster Surveys (MICS), and Performance Monitoring and Accountability 2020 surveys—have built on lessons learned over the decades, including the experiences of the World Fertility Surveys^9^ and Contraceptive Prevalence Surveys.^10^ All 3 platforms include both married and unmarried women, except in countries (largely in North Africa and South and West Asia) where only married women are included due to cultural sensitivities, and ask questions on contraceptive use and sexual activity regardless of marital status.^11^

The calculation and reporting of key family planning indicators for married women is uniform across information platforms, but varies for unmarried women. For example, take 3 key family planning data and communication groups: DHS, the Guttmacher Institute, and World Health Organization (WHO). All 3 groups frequently produce family planning-related reports, data from which are used widely for policy, programmatic, research, and advocacy purposes. DHS reports the modern contraceptive prevalence rate (mCPR) among unmarried women only among those who reported having sex in the 1 month preceding the interview.^12^ The Guttmacher Institute and recent WHO publications report mCPR among women who reported having sex in the 3 months preceding the interview.^13^^,^^14^ This Guttmacher/WHO approach aligns with the research of Dasgupta et al. that found that limiting analysis to 1 month before the interview misses a significant proportion of unmarried women who were sexually active within a wider window of time.^15^ Ultimately, these varying approaches to measuring mCPR among unmarried women result in different mCPR estimates as well as different unmet need estimates. These differing estimates have potentially significant policy and programming ramifications (e.g., size of population in need). Given these differences, coupled with the increased need to address family planning among unmarried individuals, we aimed to address the following 3 questions:

1. How do various measurement approaches, namely different durations of sexual recency, impact estimates of mCPR and unmet need among unmarried women?
2. What are the benefits and limitations of each measurement approach?
3. Is it beneficial to standardize the measurement approach? Why or why not?

Differing estimates of mCPR and unmet need among unmarried women have potentially significant policy and programming ramifications.

## DATA AND METHODS

Data came from the DHS, nationally representative household surveys that 90 countries have implemented with technical assistance from the DHS Program since 1984, when the U.S. Agency for International Development began the program. All women aged 15–49 in sampled households are eligible for the women’s interview, further details of which are elaborated upon elsewhere.^16^ Our study included DHS data that met the following 4 inclusion criteria: (1) data were collected from both married and unmarried women; (2) data were collected from 2012 or later (we chose the 2012 demarcation to complement FP2020 goals outlined in the 2012 London Summit); (3) final datasets were available for download and analysis as of February 2019; and (4) for countries that had more than 1 DHS since 2012, we included only the most recent.

Our final study dataset included 43 country surveys across 5 regions: Asia and Pacific, West Asia and Eastern Europe, East and Southern Africa, West and Central Africa, and Latin America and the Caribbean. Survey sample sizes ranged from 5,329 women in Comoros to 699,686 in India.

The key demographic variable in our analysis was marital status, unmarried (never married or formerly married) and currently married (married or in-union). We first ran our analysis by separating women into 3 groups, “never married,” “currently married or in-union,” and “formerly married (separated, divorced, or widowed).” Due to small sample sizes in the latter group, particularly when comparing sexual recency variables (described below), we decided to combine “formerly married” and “never married” into 1 group, “unmarried,” with a majority of women being represented by the “never married” group. Recent research has found that the prevalence of divorce is low, ranging from 1%–20% in sub-Saharan Africa,^17^ and the proportions are either stable or declining.^18^ In our study countries, the “unmarried” group was dominated by “never married women” who comprised 80% of all “unmarried” women, ranging from 71% in Latin America and Caribbean to 86% in Asia. Although we were aware of meaningful differences in being “never married” and “formerly married,” we combined the 2 categories due to power limitations and interpreted our results with that caveat. Additionally, we initially included age as a key demographic variable in our analysis but chose not to present the results herein as they were not particularly meaningful due to very small sample sizes by age group (tables available on request). Finally, we considered whether our marital status grouping aligned with our sexual recency variables (described below); that is, did current marital status align with sexual activity status of 1–12 months before the interview. To ensure we were using comparable categories, we analyzed marital duration at the time of survey (note: DHS does not capture data on timing of divorce). On average, only 4% of women were married for less than 12 months at the time of survey (ranging from 2% to 7% across study countries), and the overwhelming majority of these newlywed women (81%) reported sex in the last month. We decided that the potential misalignment between sexual activity recency and marital duration recency was too small to skew results; therefore, we consistently categorized “currently married” women as “married or in-union” for each of our sexual recency variables.

Our main analytic variable was timing of last sex (e.g., sexual recency). In DHS, participants are asked how old they were when they first had sexual intercourse. For those who say they have ever been sexually active, they are then asked an open-ended question, “When was the last time you had sexual intercourse?” For women who respond that they have been sexually active within the 12 months preceding the interview, the answer is recorded in days, weeks, or months. We used the variable that asked respondents what their time since last sex was in months, what we call “sexual recency,” to form 4 corresponding analytic groups:

1. DHS method (sexually active within the 4 weeks/1 month preceding the interview; note that in this article, we use the term 1 month)
2. Guttmacher Institute/WHO method (sexually active within the 3 months preceding the interview; note that the Guttmacher calculation includes months 0, 1, 2, and 3)
3. An alternative method periodically used in research (sexually active in the 12 months preceding interview)
4. All sexually active women regardless of timing of last sex (e.g., those women who have ever had sex)

We excluded incomplete or inconsistent responses. Additionally, we excluded women for whom responses were flagged for various reporting issues.^19^ For example, we treated responses that were “before last birth” as instances where the timing of last sex was more than 12 months prior.

The 2 key family planning variables are the mCPR and unmet need. We aligned our definition of modern contraceptive methods with the DHS definition, which includes female and male sterilization, contraceptive pill, intrauterine device, injectables, implants, male and female condoms, diaphragm, contraceptive foam and jelly, lactational amenorrhea method, Standard Days method, and emergency contraception.^12^ We also used the DHS definition of mCPR, which assesses contraceptive use based on the question, “Are you or your partner currently doing something or using any method to delay or avoid getting pregnant?” As Dasgupta et al. (2017) describe, the interpretation of “current” is up to the woman, ranging from whether she means today, within the last month, or at the time of last sexual intercourse.^15^

We used the DHS Program’s revised calculation of unmet need as a starting point for unmet need estimation.^20^ Unmet need is defined as the percentage of women who are not currently using a method of contraception and want to stop or delay childbearing.^20^ Typically, calculations of unmet need assume all married women are sexually active and, thus, at risk of unmet need if they are not using contraception and do not desire to become pregnant,^21^ but because unmarried women who report no sexual intercourse in the 1 month before the interview are assumed to be unexposed to the risk of pregnancy, they are considered not at risk of having unmet need.^20^ Some of these assumptions of risk have been challenged over time, and sensitivity tests demonstrate that unmet need estimates are indeed affected by the assumption used.^22^ Because the DHS calculation of unmet need among unmarried women applies the inclusion criteria of sex in the 1 month before the interview, we recalculated unmet need by relaxing the sexual activity assumptions to align with each of our sexual recency categories.

First, we explored how various sexual recency “cutoffs” impacted the proportion of women included in mCPR and unmet need estimates by marital status. These results allowed us to visualize the change in denominator (universe of eligible women) based on timing of sex. Next, we explored how estimates of mCPR and unmet need vary by timing of last sex. We also disaggregated these estimates of mCPR and unmet need by marital status. All data presented were weighted. For regional averages presented, all averages were equally weighted by country. We used Stata 14 for data analysis.

## RESULTS

### How Do Various Sexual Recency Cutoffs Impact the Proportion of Women Included in mCPR and Unmet Need Estimates?

**FIGURE 1.**
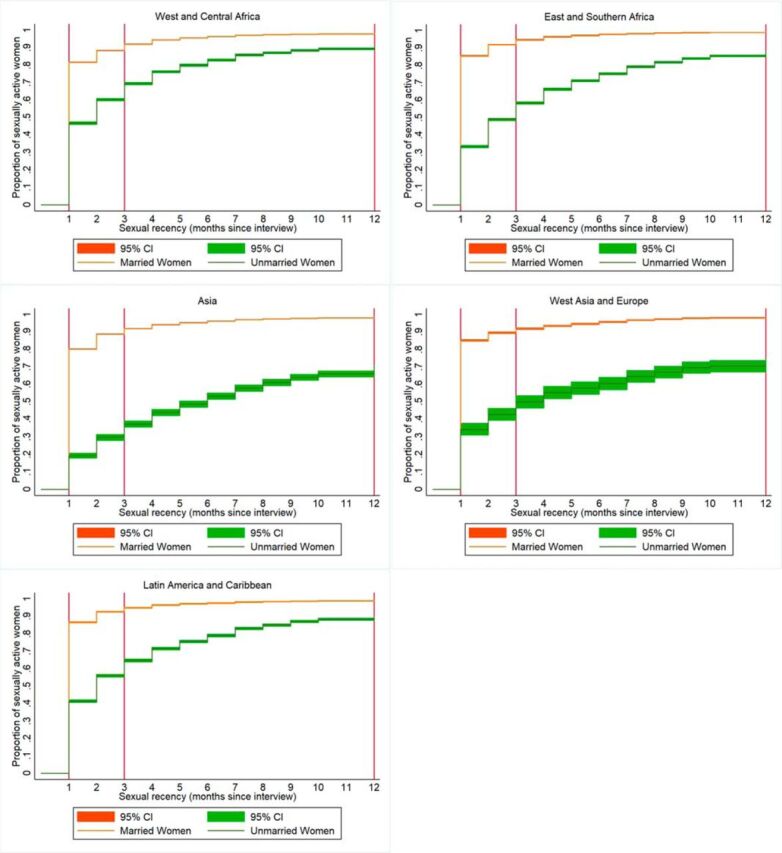
Kaplan-Meier Curves for Sexual Recency (Months Before Interview) by Region and Marital Status^a^ ^a^ Regional data presented are pooled and weighted at the country level.

The Table presents data on the variation in the number and percentage of eligible women based on the timing of sexual recency, by region and marital status. Overall, the majority of women report ever having sexual intercourse, ranging from an average of 70% of all women in the Asia/Pacific region to 83% in the West and Central Africa region. These percentages markedly decrease across all 5 regions as the sexual recency time frame narrows to sex within the last 12 months, 3 months, and finally 1 month (Figure 1). Among unmarried women, the percentage of women who report ever having sex varies dramatically by regional context, from 16% in Asia and Pacific to 62% in Latin America and Caribbean. These percentages decline as the sexual recency window narrows. At the 1-month mark, on average only 1% of unmarried women in Asia and Pacific report sexual activity. The highest percentage of unmarried women reporting sex in the last month (19%) is in West and Central Africa.

**TABLE.** Mean Percentage and Mean Number of Women Reporting Sex Within Various Time Frames, by Region and Marital Status (as Averaged by Country)

	**All Women**				
	**Total % (No.)**	**Ever Had Sex % (No.)**	**Sex in Last 12 Months % (No.)**	**Sex in Last 3 Months % (No.)**	**Sex in Last 1 Month % (No.)**
**East and Southern Africa**	100.0 (14,589)	79.2 (11,650)	64.5 (9,000)	57.4 (8,055)	46.4 (6,603)
**West and Central Africa**	100.0 (14,022)	82.3 (11,451)	69.4 (9,724)	62.7 (8,866)	51.1 (7,321)
**West Asia/Europe**	100.0 (8,976)	73.9 (6,666)	64.4 (5,777)	59.8 (5,333)	54.5 (4,823)
**Asia and Pacific**	100.0 (118,043)	70.2 (83,349)	59.0 (21,541)	54.4 (20,068)	46.3 (17,006)
**Latin America and Caribbean**	100.0 (21,409)	82.4 (17,764)	72.8 (15,631)	64.6 (13,987)	53.1 (11,551)
	**Unmarried Women**				
	**Total % (No.)**	**Ever Had Sex % (No.)**	**Sex in Last 12 Months % (No.)**	**Sex in Last 3 Months % (No.)**	**Sex in Last 1 Month % (No.)**
**East and Southern Africa**	100.0 (6,075)	58.0 (3,591)	36.3 (2,114)	24.2 (1,406)	12.4 (710)
**West and Central Africa**	100.0 (4,145)	58.2 (2,463)	42.2 (1,807)	32.0 (1,379)	19.5 (847)
**West Asia/Europe**	100.0 (2,822)	21.4 (609)	6.8 (201)	3.7 (111)	2.2 (69)
**Asia and Pacific**	100.0 (32,303)	15.9 (3,955)	4.5 (479)	1.9 (210)	0.8 (93)
**Latin America and Caribbean**	100.0 (9,505)	61.8 (5,964)	46.0 (4,430)	32.5 (3,174)	18.5 (1,838)
	**Married Women**				
	**Total % (No.)**	**Ever Had Sex % (No.)**	**Sex in Last 12 Months % (No.)**	**Sex in Last 3 Months % (No.)**	**Sex in Last 1 Month % (No.)**
**East and Southern Africa**	100.0 (8,063)	99.9 (8,059)	90.2 (6,885)	86.9 (6,649)	76.3 (5,893)
**West and Central Africa**	100.0 (8,998)	99.9 (8,988)	86.9 (7,918)	81.6 (7,487)	69.6 (6,474)
**West Asia/Europe**	100.0 (6,060)	100.0 (6,057)	93.1 (5,576)	87.7 (5,222)	80.5 (4,754)
**Asia and Pacific**	100.0 (79,507)	100.0 (79,394)	87.4 (21,063)	82.0 (19,858)	70.2 (16,914)
**Latin America and Caribbean**	100.0 (11,800)	100.0 (11,800)	95.2 (11,201)	91.5 (10,813)	81.8 (9,713)

Currently married women are more likely to have had sex recently (e.g., within the last 1 month, 3 months, and 12 months) as compared with unmarried women (Table and Figure 1). Data presented further highlight the reality that although marriage is nearly a perfect proxy for experiencing sexual intercourse at some time point (nearly 100% of married women report ever having had sex), it is not a perfect proxy for recent sexual activity. For example, across the 5 regions, on average the percentage of married women reporting sex in the 3 months preceding the interview ranges from 82%−92%. This percentage is lower for sex in the 1 month preceding the interview, where the average reporting ranges from 70%−82% of married women.

Ultimately, limiting the number of women included in mCPR and unmet need estimates to those who report sex in the 1 month preceding the interview provides analysts with sufficiently large numbers of all women and married women for complex analyses (generally more than 5,000 individuals). However, this limit can pose a challenge for analyzing the data of unmarried women, especially in countries and regions where sex outside of marriage is infrequent (or infrequently reported). In the regions of West Asia/Europe and Asia and Pacific, the 1-month cutoff yields fewer than 100 individuals on average. Expanding to those who report sex in the 3 months preceding the interview yields higher numbers in these 2 regions but still less than 250 individuals on average. For national-level estimates of mCPR and unmet need for unmarried women, these sample sizes are generally large enough. For further disaggregation and more complex analyses, these sample sizes are likely too small in many study countries.

### How Do mCPR and Unmet Need Estimates Vary by Timing of Last Sex?

**FIGURE 2.**
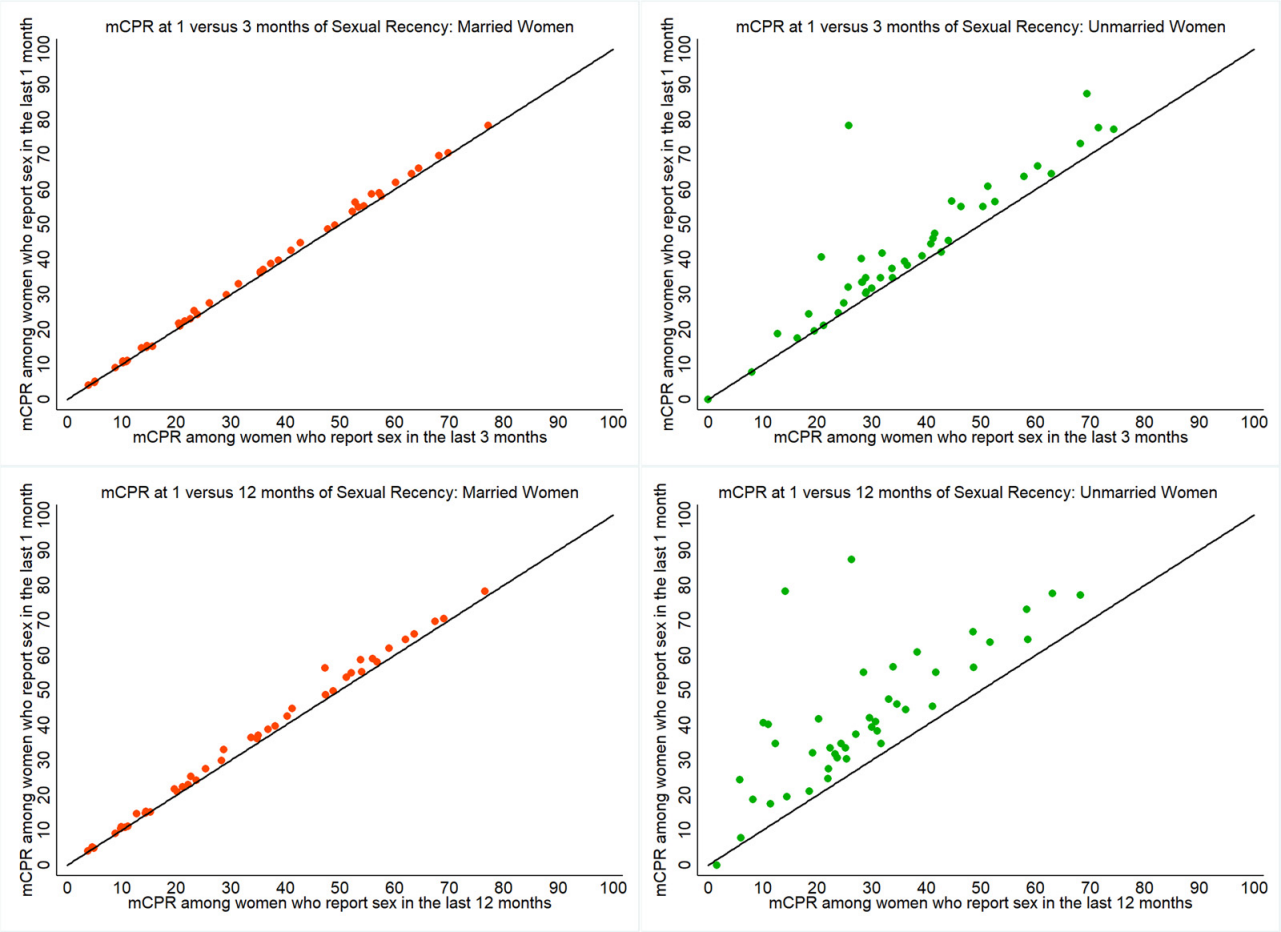
Modern Contraceptive Prevalence Rate Among Women Who Reported Sex in the Previous 1 Month Compared With Those Who Reported Sex in the Previous 3 Months and 12 Months, by Marital Status for 43 Countries Abbreviation: mCPR, modern contraceptive prevalence rate.

Applying these various sexual activity restrictions to the calculation of mCPR for all countries (Figure 2), we observe that for married women, mCPR is virtually the same as measured at the 12-month, 3-month, and 1-month eligibility cutoffs. However, for unmarried women, mCPR is systematically lower as measured at the 12-month and 3-month marks as compared with the 1-month cutoff. Indeed, mCPR as measured among unmarried women who reported sex within the 12 months preceding the interview is lower—on average, 14 percentage points lower—across all 43 study countries as compared with mCPR as measured using the 1-month criterion.

**FIGURE 3.**
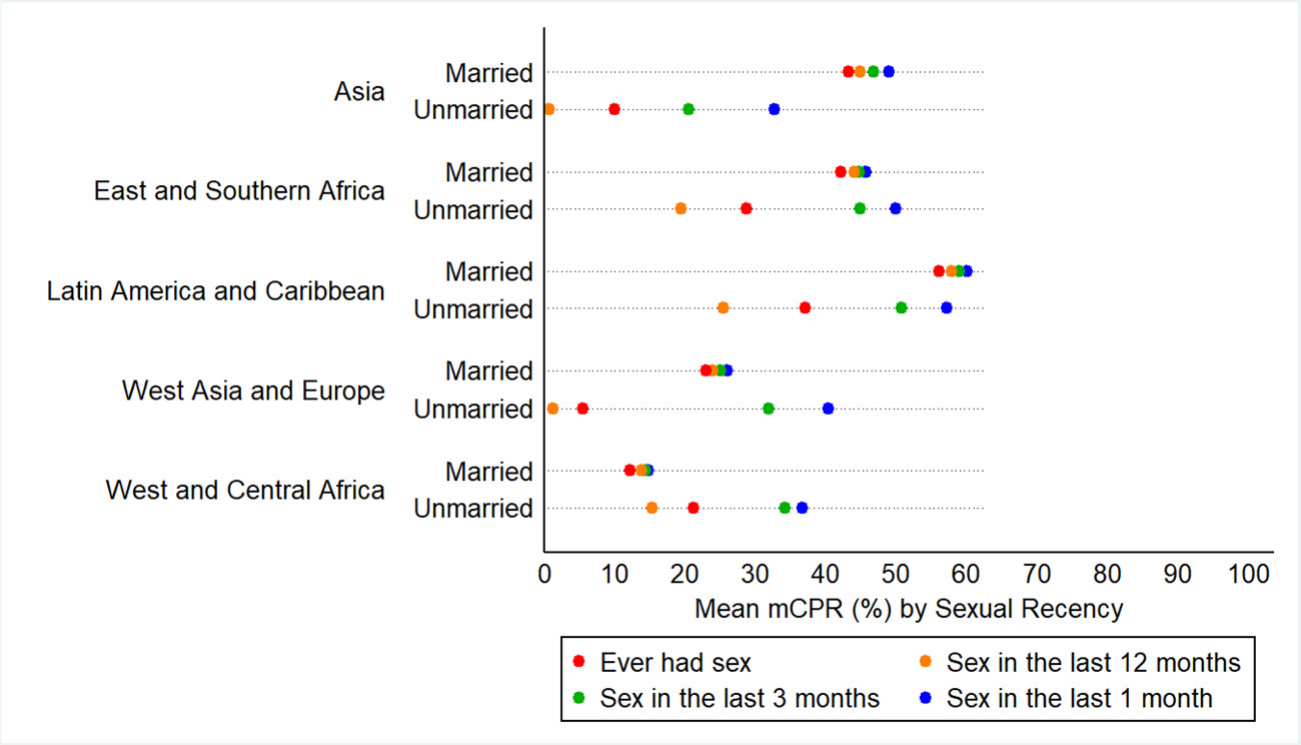
Mean Modern Contraceptive Prevalence Rate by Sexual Recency and Marital Status for Each Geographic Region Abbreviation: mCPR, modern contraceptive prevalence rate

These differences are starker when parsed out by regions (Figure 3). Among married women, we see that mCPR estimates based on the sexual recency cutoff of 1 month is virtually indistinguishable from the 3-month cutoff in all regions (varying by 1–2 percentage points). In all regions, the 1-month cutoff results in higher reports of contraceptive use regardless of marital status. Among all regions, married women who have ever been sexually active had the lowest mCPR compared to married women at the other sexual recency cutoffs. Furthermore, unmarried women who report sexual activity in the previous 12 months had the lowest mCPR among both married and unmarried women at the other sexual recency cutoffs (note: mCPR by sexual recency and marital status for all countries is presented in Supplement 1).

Next, we turn our attention to unmet need. The DHS calculation of unmet need among unmarried women applies the inclusion criteria of sex in the 1 month before the interview, which aligns with the DHS mCPR calculation among unmarried women. Because we changed the mCPR calculation among unmarried women to a broader sexual recency time frame, we needed to align our unmet need calculation. Therefore, we recalculated unmet need to expand the inclusion criteria to women who were sexually active within 3 months before the interview and 12 months before the interview versus the current DHS standard of 1 month.

**FIGURE 4.**
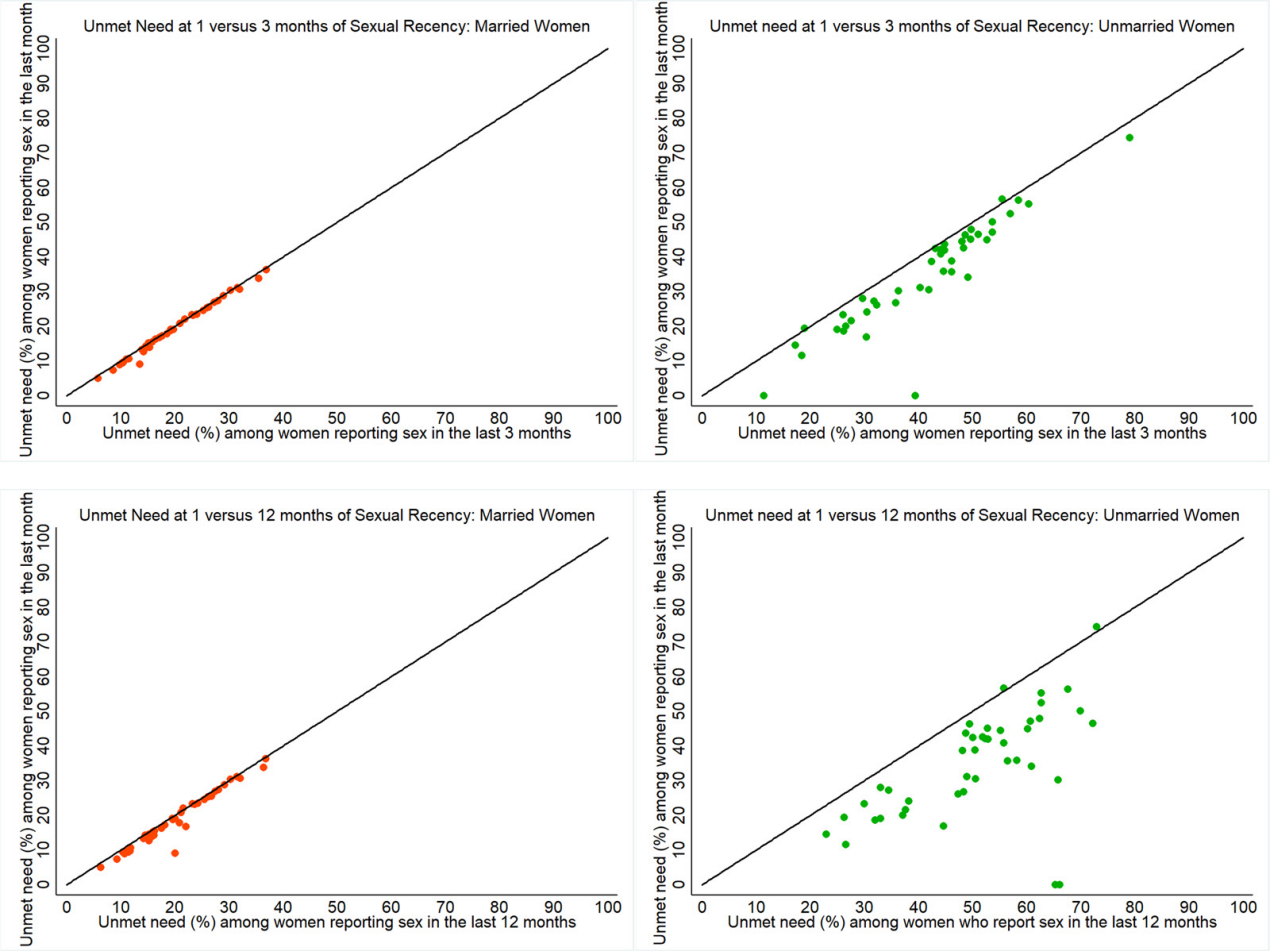
Unmet Need Among Women Who Reported Sex in the Previous 1 Month Compared With Those Who Reported Sex in the Previous 3 Months and 12 Months, by Marital Status for 43 Countries

**FIGURE 5.**
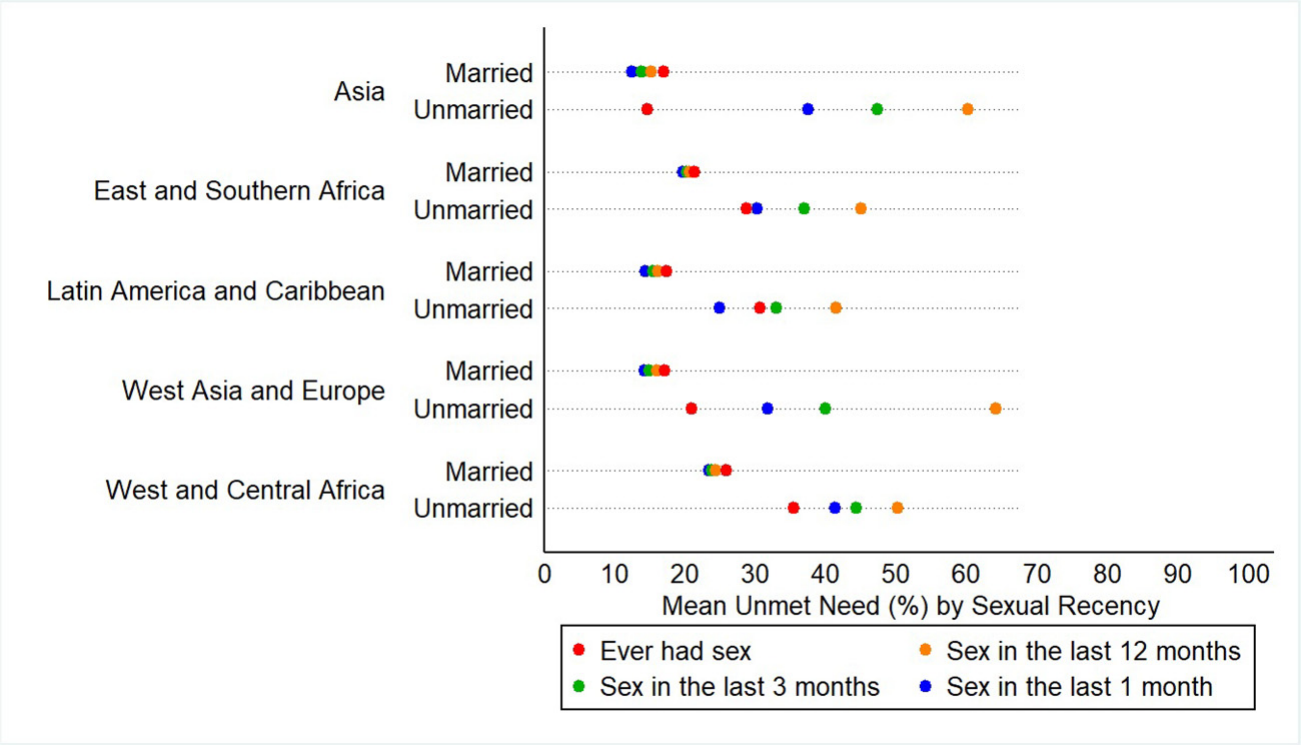
Mean Unmet Need for Family Planning by Sexual Recency and Marital Status for Each Geographic Region

As Figure 4 shows, expanding the inclusion criteria to capture unmarried women with less recent sex results in modest to large increases in unmet need. Unmet need among unmarried women across our 43 study countries is, on average, 34% (1 month), 41% (3 months), and 51% (12 months). Among married women, the difference is minimal—19% (1 month), 19% (3 months), and 20% (12 months)—as would be expected because mCPR among married women varies little by sexual recency (note: unmet need by sexual recency and marital status for all countries is presented in Supplement 2). Corresponding to the regional differences in mCPR based on the sexual recency cutoffs, the picture for unmet need shows that estimates are lowest when using the 1-month cutoff, and unmet need is highest when using the 12-month cutoff (Figure 5). Unmet need is highest among unmarried women in each of the 5 regions as compared with married women.

## IMPLICATIONS

As has been revealed and elaborated in recent scholarship, a substantial proportion of unmarried women have ever had sex.^6^^,^^23^ Our findings confirm this reality and again highlight the relatively fluid and sporadic nature of unmarried women’s sexual lives as compared with married women’s (Table). This finding may spur the measurement community to expand the eligibility criteria for mCPR and unmet need calculation to a wider pool of sexually active women (i.e., expand from inclusion criteria of sexual activity within 1 month to 3 months or 3 months to 12 months). Indeed, in so doing, we would be able to have a larger sample size for disaggregation and analysis and perhaps understand contraceptive use dynamics of a wider population of unmarried women.

Our data also reveal that expanding the eligibility range from 1 month to 3 months and beyond yields lower mCPR estimates and higher unmet need estimates. This is to be expected—not because women who had sex less recently are necessarily less likely than others to use contraception or more likely to be in greater need of contraception—but rather because expanding the inclusion criteria based on time since last sex creates a concerning measurement misalignment. Specifically, as previously mentioned, contraceptive use is based on a “current use” measure. This “current” measure aligns well with the experiences of women who had sex more recently. However, in seeking to understand contraceptive use of women whose last sex was 3 or more months ago, a reliable estimate is unlikely to be obtained based on a “current use” question.^11^^,^^24^ Coital-dependent methods are especially likely to be underreported to a “current use” question among women who are not recently sexually active. To remedy the measurement misalignment, contraceptive use at last sex would need to be examined.

In the DHS, contraceptive use at last sex can be assessed by proxy using the contraceptive calendar. A recent analysis^11^ did just that, exploring CPR among 3 groups of women: (1) women who had sex in the last 3 months, by using the contraceptive calendar to assess whether she used contraceptives in the 3 months before the interview; (2) women who had sex in the last 3 months, using the responses to the “current use” questions to define contraceptive use; and (3) women who had sex in the last 1 month, measuring contraceptive use based on responses to “current use.” The results showed that CPR was similar in groups 1 and 3, but slightly lower as measured for group 2. Based on these findings, plus the fact that contraceptive calendar data are not as widely available as current use data, the study authors chose to use group 3 as their analytic group. We agree with this assessment.

Because DHS data are cross-sectional, unmarried women who meet the 1-month inclusion criteria are likely to represent all types of sexual activity—frequent, sporadic, and periodic. Therefore, mCPR estimates and unmet need estimates reliably reflect the population-level estimates for this diverse demographic group. Of course, it is possible that there is some degree of seasonality to sexual recency, depending on cultural context, for example, with high levels of male migration. Evidence of seasonality impact on sexual activity is scarce across country contexts. Furthermore, because DHS fieldwork often spans 4 or more months, any potential seasonality impact is diminished. The major downside of this approach, as mentioned earlier, is that sample sizes of unmarried women who met the 1-month inclusion criteria can be quite small in many countries, limiting statistical power for further disaggregation and more complex analysis, especially for family planning among sexually active youth—a key population for international family planning programming. Therefore, we make 2 recommendations:

1. In the short term, we recommend maintaining the DHS method (sexual activity within the previous 1 month) for reporting mCPR and unmet need among unmarried women to more accurately monitor both indicators. For clarity and messaging, we recommend that the wider family planning community adopt this approach for measuring and reporting mCPR and unmet need among unmarried women.
2. In the longer term, we recommend adding the following 2 questions to the DHS women’s questionnaire and other surveys that capture contraceptive use data: (1) “The last time you had sex, did you or your partner do anything or use any method to avoid or delay pregnancy?” (2) [if yes] “What method did you use?” These questions align with questions already asked in the DHS men’s questionnaire as well as questions included in the U.S. National Survey of Family Growth.^25^ Incorporating these questions would help to overcome the measurement misalignment described herein, thereby allowing for improved calculation of mCPR among unmarried women who had sex less recently and allowing for analysts to use contraceptive use data for a larger number of unmarried women, thus, helping to overcome the sample size issue. For example, this approach would improve our ability to compare mCPR and unmet need estimates by marital status thanks to the greater statistical power it provides. As of editing this article in October 2019, we can report that the DHS Program has incorporated these suggested questions into its DHS-8 core women’s questionnaire.^26^ We encourage the family planning community to incorporate these new data into contraceptive prevalence and unmet need calculations as the data become available.

New DHS questions on contraceptive use at last sex can improve calculation of mCPR among unmarried women who had sex less recently.

Even when both of these recommendations came to fruition, measurement challenges remain. First, in many countries unmarried women face stigma for sexual activity outside of marriage. Such cultural sensitivity may make unmarried women unlikely to report sexual activity, leading to reporting bias.^27^^,^^28^ Looking more closely at country-specific data (Supplement 1), we observe just how low reported sexual activity rates are among unmarried women in places with large social sanctions on premarital sex. For example, in Nepal, a country with strong norms prohibiting premarital sex, only 4 unmarried women in the entire survey reported sex in the preceding month. Although disclosure of premarital sex is rare, this finding does not necessarily mean that premarital sex itself is rare. Indeed, a body of research is forming that reveals prevalence of premarital sex in Nepal is not uncommon.^29^ Some welcome survey innovations are taking place, like using audio computer assisted self interview rather than face-to-face interviews, which may reduce reporting bias by better capturing responses to sensitive questions like sexual activity and sexual history.^30^^,^^31^ More can and should be done. For example, question order and wording should be considered to reduce bias. Although DHS asks sexual activity questions much later in the questionnaire—a proven practice for building rapport before arriving at stigmatized issues like sexual behavior^32^—the sexual recency questions come immediately after marital status questions, which may unintentionally invite stigmatized responses from unmarried women in places like Nepal.

Even if we can decrease reporting bias, we still have the challenge of nonresponse and survey representativeness.^31^ Specifically, those unmarried women who are sexually active and report sexual activity may have different life experiences than other sexually active unmarried women who are uncomfortable reporting sexual activity. It is possible that unmarried women who report sexual activity are more likely to access and use contraception, which would artificially inflate mCPR among unmarried women and deflate unmet need.

Finally, because sexual activity is more likely to be sporadic among unmarried women compared with married women, it is important to recognize that these women are perhaps more likely to be unprepared (with contraception) for their next sexual encounter and more exposed to the risk of an unintended pregnancy as a result. This is a key challenge that public health programs can and should address. In this vein, it is imperative that the global family planning community commit to having solid, reliable, and comparable data to inform programming to address the family planning needs of unmarried women.

## Supplementary Material

19-00298-Fabic-Supplement2.docx

19-00298-Fabic-Supplement1.docx

## Notes

**Peer Reviewed**

## References

[1] Brown W, Druce N, Bunting J, et al. Developing the “120 by 20” goal for the Global FP2020 Initiative. Stud Fam Plann. 2014;45(1):73–84. 10.1111/j.1728-4465.2014.00377.x. 24615576

[2] Cahill N, Sonneveldt E, Stover J, et al. Modern contraceptive use, unmet need, and demand satisfied among women of reproductive age who are married or in a union in the focus countries of the Family Planning 2020 initiative: a systematic analysis using the Family Planning Estimation Tool. Lancet. 2018;391(10123): 870–882. 10.1016/S0140-6736(17)33104-5. 29217374 PMC5854461

[3] Darroch JE, Singh S. Trends in contraceptive need and use in developing countries in 2003, 2008, and 2012: an analysis of national surveys. Lancet. 2013;381(9879):1756–1762. 10.1016/S0140-6736(13)60597-8. 23683642

[4] Sathar Z, Chidambaram VC. *Differentials in Contraceptive Use. WFS Comparative Studies 36*. Voorburg, Netherlands: International Statistical Institute; 1984.

[5] *Population Facts: World Marriage Patterns*. 2011/11. New York, NY: United Nations Department of Economic and Social Affairs, Population Division; 2011.

[6] MacQuarrie KLD, Mallick L, Allen C. *Sexual and Reproductive Health in Early and Later Adolescence: DHS Data on Youth Age 10–19*. DHS Comparative Reports No. 45. Rockville, MD: ICF; 2017. http://dhsprogram.com/pubs/pdf/CR45/CR45.pdf.

[7] Das Gupta M, Engelman R, Levy J, Luchsinger F, Merrick T, Rosen JE. *The Power of 1.8 Billion: Adolescents, Youth and the Transformation of the Future*. State of World Population 2014. New York, NY: UNFPA; 2014. https://www.unfpa.org/sites/default/files/pub-pdf/EN-SWOP14-Report_FINAL-web.pdf. Accessed May 24, 2019.

[8] Rimon JG, Tsui AO. Regaining momentum in family planning. Glob Health Sci Pract. 2018;6(4):626–628. 10.9745/GHSP-D-18-00483. 30591572 PMC6370364

[9] Kendall MG. *The World Fertility Survey: Current Status and Findings. Population Reports* M(3). Voorburg, Netherlands: International Statistical Institute; 1979. 514924

[10] Morris L, Lewis G, Powell D, et al. *Contraceptive Prevalence Surveys: A New Source of Family Planning Data. Population Reports M(5)*; 1981. 6973767

[11] Wang W, Staveteig S, Winter R, Allen C. *Women’s marital status, contraceptive use, and unmet need in Sub-Saharan Africa, Latin America, and the Caribbean*. DHS Comparative Report No. 44. Rockville, Maryland: ICF; 2017. http://dhsprogram.com/pubs/pdf/CR44/CR44.pdf. Accessed May 24, 2019.

[12] Croft TN, Marshall AMJ, Allen CK. *Guide to DHS Statistics*. Rockville, MD: ICF; 2018.

[13] Darroch J. Adding It Up: *Investing in Contraception and Maternal and Newborn Health, 2017 - Estimation Methodology*. New York, NY: Guttmacher Institute; 2018.

[14] Hindin MJ, Kalamar AM. Country-specific data on the contraceptive needs of adolescents. Bull World Health Organ. 2017;95(3):166. 10.2471/BLT.16.189829. 28250525 PMC5328115

[15] Dasgupta A, Ueffing P, Kantorová V. *Sexual Activity by Marital Status and Age: A Comparative Perspective. United Nations, Department of Economic and Social Affairs, Population Division* Technical Paper No. 11. New York, NY: United Nations; 2017.

[16] Fabic MS, Choi Y, Bird S. A systematic review of Demographic and Health Surveys: data availability and utilization for research. Bull World Health Organ. 2012;90:604–612. 22893744 10.2471/BLT.11.095513 PMC3417790

[17] Smith-Greenaway E, Clark S. Variation in the link between parental divorce and children’s health disadvantage in low and high divorce settings. SSM Popul Health. 2017;3:473–486. 10.1016/j.ssmph.2017.04.004. 28890915 PMC5589346

[18] Clark S, Brauner-Otto S. Divorce in sub-Saharan Africa: are unions becoming less stable? Popul Development Review. 2015;41(4):583–605.

[19] Heger Boyle E, King M, Sobek M. *Data Description- TIMESINCESEXFLAG: IPUMS-Demographic and Health Surveys*: Version 7. Minnesota Population Center and ICF International; 2019. https://www.idhsdata.org/idhs-action/variables/TIMESINCESEXFLAG#description_section.

[20] Bradley SEK, Croft TN, Fishel JD, Westoff CF. *Revising Unmet Need for Family Planning*. DHS Analytical Studies No. 25. Calverton, MD: ICF; 2012. https://dhsprogram.com/pubs/pdf/AS25/AS25[12June2012].pdf.

[21] MacQuarrie KLD. *Marriage and Fertility Dynamics: the Influence of Marriage Age on the Timing of First Birth and Birth Spacing*. DHS Analytical Studies No. 56. Rockville, MD: ICF; 2016. http://dhsprogram.com/pubs/pdf/AS56/AS56.pdf.

[22] Bradley SE, Casterline JB. Understanding unmet need: history, theory, and measurement. Stud Fam Plann. 2014;45(2):123–150. 10.1111/j.1728-4465.2014.00381.x. 24931072 PMC4369378

[23] Kalamar AM, Tunçalp Ö, Hindin MJ. Developing strategies to address contraceptive needs of adolescents: exploring patterns of use among sexually active adolescents in 46 low- and middle-income countries. Contraception. 2018;98(1):36–40. 10.1016/j.contraception.2018.03.016. 29550455

[24] Fabic MS, Becker S. Measuring contraceptive prevalence among women who are at risk of pregnancy. Contraception. 2017;96(3):183–188. 10.1016/j.contraception.2017.06.007. 28666794

[25] Aiken AR, Wang Y, Higgins J, Trussell J. Similarities and differences in contraceptive use reported by women and men in the National Survey of Family Growth. Contraception. 2017;95(4):419–423. 10.1016/j.contraception.2016.10.008. 27823945 PMC5376522

[26] Demographic and Health Survey (DHS) Program. DHS Model Questionnaires: Phase 8. Washington, DC: DHS; 2019. https://dhsprogram.com/publications/publication-DHSQ8-DHS-Questionnaires-and-Manuals.cfm. Accessed October 25, 2019.

[27] Beguy D, Kabiru CW, Nderu EN, Ngware MW. Inconsistencies in self-reporting of sexual activity among young people in Nairobi, Kenya. J Adolesc Health. 2009;45(6):595–601. 10.1016/j.jadohealth.2009.03.014. 19931832 PMC2784947

[28] Kelly CA, Soler-Hampejsek E, Mensch BS, Hewett PC. Social desirability bias in sexual behavior reporting: evidence from an interview mode experiment in rural Malawi. Int Perspect Sex Reprod Health. 2013;39(1):14–21. 10.1363/3901413. 23584464 PMC4023461

[29] Adhikari R, Tamang J. Premarital sexual behavior among male college students of Kathmandu, Nepal. BMC Public Health. 2009;9(1):241. 10.1186/1471-2458-9-241. 19604383 PMC2717085

[30] Hewett PC, Mensch BS, Erulkar AS. Consistency in the reporting of sexual behaviour by adolescent girls in Kenya: a comparison of interviewing methods. Sex Transm Infect. 2004;80(Suppl 2):ii43–ii48. 10.1136/sti.2004.013250. 15572639 PMC1765856

[31] Langhaug LF, Sherr L, Cowan FM. How to improve the validity of sexual behaviour reporting: systematic review of questionnaire delivery modes in developing countries. Trop Med Int Health. 2010;15(3):362–381. 10.1111/j.1365-3156.2009.02464.x 20409291 PMC3321435

[32] Fenton KA, Johnson AM, McManus S, Erens B. Measuring sexual behaviour: methodological challenges in survey research. Sex Transm Infect. 2001;77(2):84–92. 10.1136/sti.77.2.84. 11287683 PMC1744273

