# Supplementary material for: Standardizing Measurement of Contraceptive Use Among Unmarried Women
Source: Glob Health Sci Pract. 2019 Dec 23;7(4):564–74. doi: 10.9745/GHSP-D-19-00298 (PMC6927838; doi:10.9745/GHSP-D-19-00298)
Supplement: 19-00298-Fabic-Supplement2.docx [file 19-00298-Fabic-Supplement2.docx]

**19-00298-Fabic Supplement 2. Proportion of Women (aged 15−49) with Unmet Need for Family Planning by Sexual Recency and Marital Status, for All Study Countries**

|  | **Married women** | | | |  | **Unmarried women** | | | |  | **All women** | | | |
| --- | --- | --- | --- | --- | --- | --- | --- | --- | --- | --- | --- | --- | --- | --- |
|  | **Ever had sex**  **% (No.)** | **12 months**  **% (No.)** | **3 months**  **% (No.)** | **1 months % (No.)** |  | **Ever had sex**  **% (No.)** | **12 months % (No.)** | **3 months % (No.)** | **1 months**  **% (No.)** |  | **Ever had sex % (No.)** | **12 months % (No.)** | **3 months**  **% (No.)** | **1 months % (No.)** |
| **East and Southern Africa** | | | |  |  |  |  |  |  |  |  |  |  |  |
| Angola (2015-16) | 38.2 | 36.8 | 36.9 | 36.3 |  | 39.3 | 51.9 | 48.3 | 42.6 |  | 38.6 | 41.9 | 40.2 | 37.7 |
|  | (7,402) | (6,155) | (5,916) | (5,009) |  | (4,867) | (3,145) | (2,460) | 1,483 |  | (12,269) | (9,300) | (8,376) | (6,492) |
| Burundi (2016-17) | 29.8 | 29.2 | 29.0 | 28.8 |  | 24.4 | 56.5 | 46.2 | 35.6 |  | 28.7 | 31.5 | 29.8 | 28.9 |
|  | (9,436) | (8,842) | (8,648) | (7,860) |  | (2,427) | (836) | (417) | 146 |  | (11,863) | (9,678) | (9,065) | (8,006) |
| Comoros (2012) | 31.1 | 30.3 | 30.3 | 30.4 |  | 31.6 | 50.6 | 42.0 | 30.6 |  | 31.1 | 32.0 | 30.9 | 30.4 |
|  | (2,934) | (2,931) | (2,771) | (2,409) |  | (439) | (288) | (165) | 94 |  | (3,373) | (3,219) | (2,936) | (2,503) |
| Ethiopia (2016) | 22.3 | 21.2 | 20.9 | 20.7 |  | 21.1 | 47.4 | 32.3 | 26.1 |  | 22.1 | 23.1 | 21.4 | 20.8 |
|  | (9,603) | (8,531) | (8,158) | (7,114) |  | (2,106) | (856) | (414) | 186 |  | (11,709) | (9,387) | (8,572) | (7,300) |
| Kenya (2014) | 8.1 | 16.1 | 15.1 | 14.2 |  | 16.6 | 48.4 | 35.8 | 26.7 |  | 10.8 | 23.6 | 18.4 | 15.1 |
|  | (18,010) | (8,300) | (7,964) | (6,845) |  | (7,135) | (2,142) | (1,265) | 513 |  | (25,145) | (10,442) | (9,229) | (7,358) |
| Lesotho (2014) | 18.4 | 15.6 | 15.4 | 14.0 |  | 27.3 | 33.0 | 25.0 | 19.0 |  | 21.7 | 21.3 | 17.8 | 14.8 |
|  | (3,555) | (3,180) | (2,929) | (2,168) |  | (2,065) | (1,553) | (980) | 423 |  | (5,620) | (4,733) | (3,909) | (2,591) |
| Malawi (2015-16) | 18.6 | 15.9 | 14.8 | 13.9 |  | 35.0 | 50.5 | 46.1 | 38.8 |  | 22.8 | 22.4 | 18.8 | 15.4 |
|  | (15,432) | (14,006) | (13,481) | (12,169) |  | (5,489) | (3,298) | (1,945) | 787 |  | (20,921) | (17,304) | (15,426) | (12,956) |
| Namibia (2013) | 17.6 | 16.2 | 15.9 | 15.5 |  | 20.4 | 23.0 | 17.2 | 14.5 |  | 19.3 | 20.0 | 16.5 | 15.2 |
|  | (3,034) | (2,965) | (2,850) | (2,405) |  | (4,351) | (3,306) | (2,421) | 1,321 |  | (7,385) | (6,271) | (5,271) | (3,726) |
| Rwanda (2014-15) | 19.0 | 18.2 | 17.7 | 17.2 |  | 31.1 | 60.7 | 53.7 | 47.1 |  | 22.6 | 24.9 | 21.0 | 18.5 |
|  | (6,771) | (6,669) | (6,558) | (6,254) |  | (2,960) | (1,324) | (705) | 315 |  | (9,731) | (7,993) | (7,263) | (6,569) |
| South Africa (2016) | 14.6 | 14.6 | 14.6 | 14.3 |  | 24.1 | 30.0 | 26.0 | 23.2 |  | 20.3 | 23.1 | 20.4 | 18.1 |
|  | (2,722) | (2,599) | (2,513) | (2,163) |  | (4,509) | (3,503) | (2,829) | 1,732 |  | (7,231) | (6,102) | (5,342) | (3,895) |
| Tanzania (2015-16) | 22.0 | 20.1 | 19.7 | 19.0 |  | 32.1 | 38.3 | 30.5 | 24.2 |  | 24.9 | 24.7 | 21.8 | 19.7 |
|  | (8,123) | (7,437) | (7,256) | (6,621) |  | (3,081) | (2,216) | (1,532) | 797 |  | (11,204) | (9,653) | (8,788) | (,418) |
| Uganda (2016) | 28.2 | 26.8 | 26.3 | 25.4 |  | 36.5 | 49.0 | 40.4 | 31.2 |  | 30.6 | 31.9 | 28.6 | 26.0 |
|  | (11,105) | (10,115) | (9,727) | (8,463) |  | (4,358) | (2,889) | (1,885) | 861 |  | (15,463) | (13,004) | (11,612) | (9,324) |
| Zambia (2013-14) | 21.2 | 19.6 | 19.3 | 18.7 |  | 38.5 | 55.2 | 48.1 | 44.5 |  | 26.7 | 27.6 | 23.9 | 21.1 |
|  | (8,730) | (8,904) | (8,761) | (8,084) |  | (4,381) | (2,806) | (1,802) | 886 |  | (13,111) | (11,710) | (10,563) | (8,970) |
| Zimbabwe (2015) | 10.4 | 9.3 | 8.6 | 7.3 |  | 25.7 | 37.1 | 26.6 | 20.1 |  | 14.2 | 14.2 | 10.7 | 8.1 |
|  | (5,971) | (5,762) | (5,557) | (4,941) |  | (2,104) | (1,435) | (859) | 395 |  | (8,075) | (7,197) | (6,416) | (5,336) |
| **West and Central Africa** | | |  |  |  |  |  |  |  |  |  |  |  |  |
| Benin (2011-12) | 32.3 | 31.5 | 31.6 | 31.1 |  | 37.9 | 52.8 | 49.6 | 45.1 |  | 33.5 | 35.9 | 34.8 | 33.0 |
|  | (10,265) | (7,489) | (7,125) | (5,840) |  | 2,666) | (1,831) | (1,478) | 869 |  | (12,931) | (9,320) | (8,603) | (6,709) |
| Chad (2014-15) | 23.0 | 23.3 | 23.2 | 23.3 |  | 30.0 | 55.8 | 55.5 | 56.6 |  | 23.9 | 26.0 | 24.8 | 24.5 |
|  | (12,128) | (11,833) | (11,258) | (10,034) |  | (1,786) | (941) | (521) | 313 |  | (13,914) | (12,774) | (11,779) | (10,347) |
| Democratic Republic of Congo (2013-14) | 27.8 | 26.6 | 26.2 | 25.5 |  | 39.6 | 50.0 | 43.2 | 42.4 |  | 31.0 | 31.9 | 29.2 | 27.7 |
|  | (11,937) | (10,475) | (10,092) | (8,972) |  | (4,083) | (2,795) | (2,038) | 1,267 |  | (16,020) | (13,270) | (12,130) | (10,239) |
| Côte d’Ivoire (2011-12) | 27.1 | 25.5 | 25.3 | 24.5 |  | 42.2 | 48.7 | 44.9 | 43.7 |  | 32.0 | 32.6 | 30.6 | 28.7 |
|  | (5,976) | (5,275) | (5,040) | (4,261) |  | (2,672) | (2,167) | (1,741) | 1,119 |  | (8,648) | (7,442) | (6,781) | (5,380) |
| Gabon (2012) | 26.2 | 23.7 | 23.4 | 23.2 |  | 29.6 | 33.0 | 29.7 | 28.0 |  | 27.7 | 27.5 | 25.7 | 24.7 |
|  | (4,418) | (4,079) | (3,906) | (3,281) |  | (2,893) | (2,377) | (1,931) | 1,237 |  | (7,311) | (6,456) | (5,837) | (4,518) |
| Gambia (2013) | 25.1 | 24.2 | 24.1 | 23.5 |  | 28.3 | 55.8 | 44.2 | 40.9 |  | 25.5 | 26.2 | 24.9 | 23.8 |
|  | (6,408) | (5,216) | (4,847) | (4,100) |  | (732) | (331) | (169) | 73 |  | (7,140) | (5,547) | (5,016) | (4,173) |
| Ghana (2014) | 29.9 | 27.5 | 27.3 | 26.9 |  | 40.4 | 52.4 | 44.1 | 42.1 |  | 33.7 | 35.4 | 31.4 | 29.6 |
|  | (5,186) | (4,476) | (4,193) | (3,292) |  | (2,631) | (1,924) | (1,262) | 653 |  | (7,817) | (6,400) | (5,455) | (3,945) |
| Guinea (2012) | 23.7 | 22.1 | 17.2 | 16.8 |  | 43.9 | 52.9 | 44.9 | 41.9 |  | 27.0 | 27.1 | 21.3 | 19.4 |
|  | (6,552) | (5,252) | (4,121) | (3,364) |  | (1,252) | (969) | (675) | 363 |  | (7,804) | (6,221) | (4,796) | (3,727) |
| Liberia (2013) | 31.2 | 28.1 | 28.0 | 27.4 |  | 43.3 | 49.5 | 48.6 | 46.4 |  | 35.8 | 35.9 | 35.0 | 33.0 |
|  | (5,545) | (4,720) | (4,567) | (3,872) |  | (2,742) | (2,107) | (1,813) | 1,237 |  | (8,287) | (6,827) | (6,380) | (5,109) |
| Mali (2012-13) | 26.0 | 26.2 | 26.0 | 25.4 |  | 42.4 | 62.7 | 60.4 | 55.2 |  | 27.3 | 28.3 | 27.7 | 26.3 |
|  | (7,577) | (6,721) | (6,502) | (5,881) |  | (682) | (446) | (347) | 196 |  | (8,259) | (7,167) | (6,849) | (6,077) |
| Niger (2012) | 16.1 | 15.9 | 15.1 | 15.1 |  | 24.4 | 69.9 | 53.7 | 50.1 |  | 16.4 | 16.8 | 15.4 | 15.2 |
|  | (9,024) | (8,673) | (7,939) | (6,926) |  | (498) | (182) | (67) | 25 |  | (9,522) | (8,855) | (8,006) | (6,951) |
| Nigeria (2013) | 15.9 | 15.2 | 15.1 | 14.6 |  | 27.8 | 37.6 | 27.6 | 21.6 |  | 17.9 | 18.2 | 16.3 | 15.1 |
|  | (25,376) | (23,929) | (23,188) | (20,706) |  | (5,699) | (4,116) | (2,996) | 1,783 |  | (31,075) | (28,045) | (26,184) | (22,489) |
| Sierra Leone (2013) | 24.6 | 19.5 | 19.3 | 19.1 |  | 30.2 | 34.5 | 31.8 | 27.3 |  | 26.2 | 24.3 | 23.0 | 21.1 |
|  | (9,494) | (7,471) | (7,155) | (6,264) |  | (4,187) | (3,624) | (3,206) | 2,117 |  | (13,681) | (11,095) | (10,361) | (8,381) |
| Togo (2013-14) | 33.5 | 32.1 | 32.0 | 30.7 |  | 37.4 | 48.2 | 42.4 | 38.7 |  | 34.5 | 35.8 | 33.9 | 31.8 |
|  | (5,947) | (5,236) | (4,884) | (3,849) |  | (1,963) | (1,482) | (1,062) | 600 |  | (7,910) | (6,718) | (5,946) | (4,449) |
| **West Asia/Europe** | | |  |  |  |  |  |  |  |  |  |  |  |  |
| Albania (2017-18) | 15.1 | 14.2 | 14.0 | 13.3 |  | 36.3 | 58.2 | 44.6 | 35.9 |  | 17.6 | 17.7 | 15.8 | 14.5 |
|  | (7,474) | (6,992) | (6,779) | (6,047) |  | (684) | (384) | (268) | 193 |  | (8,158) | (7,376) | (7,047) | (6,240) |
| Armenia (2015-16) | 12.5 | 11.7 | 10.5 | 9.6 |  | 11.0 | 66.2 | 11.5 | 0.0 |  | 12.4 | 12.6 | 10.6 | 9.6 |
|  | (3,998) | (3,888) | (3,756) | (3,577) |  | (379) | (56) | (20) | 11 |  | (4,377) | (3,944) | (3,776) | (3,588) |
| Kyrgyz Republic (2012) | 18.1 | 17.6 | 16.5 | 16.3 |  | 25.1 | 60.2 | 52.7 | 44.8 |  | 19.0 | 20.3 | 17.8 | 16.9 |
|  | (5,258) | (5,148) | (4,964) | (4,674) |  | (663) | (254) | (126) | 59 |  | (5,921) | (5,402) | (5,090) | (4,733) |
| Tajikistan (2017) | 22.8 | 20.9 | 18.6 | 17.8 |  | 11.7 | 72.3 | 51.0 | 46.5 |  | 21.9 | 21.7 | 18.7 | 17.9 |
|  | (7,499) | (6,275) | (5,388) | (4,718) |  | (710) | (110) | (31) | 12 |  | (8,209) | (6,385) | (5,419) | (4,730) |
| **Asia and Pacific** | | |  |  |  |  |  |  |  |  |  |  |  |  |
| Cambodia (2014) | 12.4 | 11.5 | 11.0 | 10.4 |  | 13.1 | 65.8 | 36.4 | 30.2 |  | 12.4 | 12.7 | 11.2 | 10.5 |
|  | (11,246) | (11,006) | (10,725) | (9,651) |  | (1,266) | (286) | (102) | (50 |  | (12,512) | (11,292) | (10,827) | (9,701) |
| India (2015-16) | 13.0 | 11.8 | 11.5 | 10.7 |  | 1.0 | 26.3 | 18.9 | 19.4 |  | 12.5 | 12.1 | 11.5 | 10.7 |
|  | (473,658) | (72,161) | (67,641) | (56,771) |  | (20,273) | (1,215) | (578) | (247 |  | (493,931) | (73,376) | (68,219) | 57,018) |
| Indonesia (2012) | 11.3 | 10.8 | 9.9 | 8.9 |  | 14.8 | 67.6 | 58.5 | 56.4 |  | 11.6 | 11.6 | 10.1 | 8.9 |
|  | (30,769) | (30,287) | (29,397) | (25,730) |  | (2,241) | 573) | (185) | (71 |  | (33,010) | (30,860) | (29,582) | (25,801) |
| Myanmar (2015-16) | 16.3 | 15.4 | 14.4 | 13.4 |  | 13.2 | 60.9 | 49.2 | 34.2 |  | 15.9 | 16.5 | 14.6 | 13.5 |
|  | (7,639) | (7,112) | (6,727) | (5,804) |  | (854) | (185) | (53) | (19) |  | (8,493) | (7,297) | (6,780) | (5,823) |
| Nepal (2016) | 23.7 | 20.0 | 13.5 | 9.1 |  | 12.8 | 65.3 | 39.5 | 0.0 |  | 23.3 | 20.4 | 13.6 | 9.1 |
|  | (9,860) | (8,672) | (7,322) | (6,085) |  | (347) | (68) | (16) | (4) |  | (10,207) | (8,740) | (7,338) | (6,089) |
| Philippines (2017) | 16.7 | 15.2 | 14.3 | 12.7 |  | 26.2 | 62.4 | 49.7 | 47.8 |  | 18.1 | 18.4 | 15.7 | 13.5 |
|  | (15,159) | (13,526) | (12,763) | (10,701) |  | (2,247) | (874) | (453) | (224) |  | (17,406) | (14,400) | (13,216) | (10,925) |
| Timor Leste (2016) | 25.3 | 21.5 | 21.9 | 22.0 |  | 21.8 | 72.9 | 79.0 | 74.4 |  | 25.1 | 23.0 | 22.8 | 22.5 |
|  | (7,424) | (4,675) | (4,433) | (3,653) |  | (458) | (150) | (84) | (35) |  | (7,882) | (4,825) | (4,517) | (3,688) |
| **Latin America and Caribbean** | | | |  |  |  |  |  |  |  |  |  |  |  |
| Colombia (2015) | 6.7 | 6.3 | 5.8 | 5.0 |  | 22.5 | 26.6 | 18.5 | 11.6 |  | 13.1 | 13.8 | 9.7 | 6.6 |
|  | (19,753) | (19,142) | (18,719) | (16,918) |  | (12,387) | (10,105) | (7,597) | (4,594) |  | (32,140) | (29,247) | (26,316) | (21,512) |
| Dominican Republic (2013) | 11.0 | 10.5 | 10.2 | 9.3 |  | 26.6 | 32.0 | 26.2 | 18.6 |  | 16.8 | 17.4 | 14.4 | 11.1 |
|  | (5,054) | (5,071) | (4,900) | (4,497) |  | (2,774) | (2,226) | (1,580) | (920) |  | (7,828) | (7,297) | (6,480) | (5,417) |
| Guatemala (2014-15) | 14.0 | 11.4 | 10.3 | 9.4 |  | 24.4 | 44.6 | 30.4 | 16.9 |  | 16.4 | 16.0 | 12.2 | 9.9 |
|  | (14,840) | (13,699) | (13,190) | (12,033) |  | (4,403) | (2,086) | (1,288) | (697) |  | (19,243) | (15,785) | (14,478) | (12,730) |
| Haiti (2016-17) | 38.0 | 36.4 | 35.5 | 33.8 |  | 49.3 | 62.7 | 57.0 | 52.4 |  | 42.3 | 45.3 | 41.3 | 37.4 |
|  | (7,554) | (6,891) | (6,443) | (5,402) |  | (4,291) | (3,304) | (2,232) | (1,142) |  | (11,845) | (10,195) | (8,675) | (6,544) |
