# Supplementary material for: Standardizing Measurement of Contraceptive Use Among Unmarried Women
Source: Glob Health Sci Pract. 2019 Dec 23;7(4):564–74. doi: 10.9745/GHSP-D-19-00298 (PMC6927838; doi:10.9745/GHSP-D-19-00298)
Supplement: 19-00298-Fabic-Supplement1.docx [file 19-00298-Fabic-Supplement1.docx]

**19-00298- Supplement 1. Women (aged 15−49) Using a Modern Method of Contraception by Sexual Recency and Marital Status, for All Study Countries**

|  | **Married women** | | | |  | **Unmarried women** | | | |  | **All women** | | | |
| --- | --- | --- | --- | --- | --- | --- | --- | --- | --- | --- | --- | --- | --- | --- |
|  | **Ever had sex**  **% (No.)** | **12 months**  **% (No.)** | **3 months**  **% (No.)** | **1 months**  **% (No.)** |  | **Ever had sex**  **% (No.)** | **12 months**  **% (No.)** | **3 months**  **% (No.)** | **1 months**  **% (No.)** |  | **Ever had sex**  **% (No.)** | **12 months**  **% (No.)** | **3 months**  **% (No.)** | **1 months**  **% (No.)** |
| **East and Southern Africa** | | | | |  |  |  |  |  |  |  |  |  |  |
| Angola (2015-16) | 12.8  (7,402) | 14.2  (6,321) | 14.6  (5,916) | 14.9  (5,009) |  | 16.2  (4,867) | 14.9  (4,547) | 24.9  (2,460) | 27.5  (1,483) |  | 14.1  (12,269) | 14.5  (10,868) | 17.6  (8,376) | 17.6  (6,492) |
| Burundi (2016-17) | 22.9  (9,436) | 23.7  (8,897) | 23.8  (8,648) | 24.2  (7,860) |  | 10.9  (2,427) | 3.3  (6,116) | 28.3  (417) | 33.4  (146) |  | 20.5  (11,863) | 15.7  (15,013) | 24.0  (9,065) | 24.3  (8,006) |
| Comoros (2012) | 14.6  (2,934) | 14.9  (3,013) | 15.7  (2,771) | 15.2  (2,409) |  | 14.4  (439) | 2.8  (1,870) | 25.7  (165) | 32.1  (94) |  | 14.6  (3,373) | 10.2  (4,883) | 16.2  (2,936) | 15.8  (2,503) |
| Ethiopia (2016) | 35.3  (9,603) | 38.1  (8,572) | 38.7  (8,158) | 39.8  (7,114) |  | 15.7  (2,106) | 5.0  (4,604) | 46.4  (414) | 55.0  (186) |  | 32.4  (11,709) | 27.3  (13,176) | 39.0  (8,572) | 40.1  (7,300) |
| Kenya (2014) | 53.1  (18,010) | 55.9  (8,884) | 57.2  (7,964) | 59.0  (6,845) |  | 28.0  (7,135) | 14.3  (6,974) | 51.3  (1,265) | 60.8  (513) |  | 45.4  (25,145) | 37.2  (15,858) | 56.2  (9,229) | 59.1  (7,358) |
| Lesotho (2014) | 59.7  (3,555) | 63.5  (3,189) | 64.4  (2,929) | 66.0  (2,168) |  | 49.9  (2,065) | 37.3  (2,490) | 68.3  (980) | 73.1  (423) |  | 56.1  (5,620) | 52.1  (5,679) | 65.4  (3,909) | 67.2  (2,591) |
| Malawi (2015-16) | 58.2  (15,432) | 61.9  (14,128) | 63.1  (13,481) | 64.5  (12,169) |  | 30.5  (5,489) | 19.7  (6,363) | 40.8  (1,945) | 44.4  (787) |  | 51.0  (20,921) | 49.0  (20,491) | 60.3  (15,426) | 63.3  (12,956) |
| Namibia (2013) | 55.1  (3,034) | 56.8  (3,024) | 57.6  (2,850) | 58.1  (2,405) |  | 58.8  (4,351) | 50.1  (4,594) | 74.4  (2,421) | 77.2  (1,321) |  | 57.4  (7,385) | 52.6  (7,618) | 65.8  (5,271) | 65.4  (3,726) |
| Rwanda (2014-15) | 47.5  (6,771) | 48.8  (6,673) | 49.1  (6,558) | 49.7  (6,254) |  | 14.4  (2,960) | 6.6  (4,953) | 28.9  (705) | 34.7  (315) |  | 37.6  (9,731) | 31.1  (11,626) | 47.2  (7,263) | 49.0  (6,569) |
| South Africa (2016) | 54.2  (2,722) | 53.9  (2,630) | 54.4  (2,513) | 55.2  (2,163) |  | 54.1  (4,509) | 45.8  (4,595) | 62.9  (2,829) | 64.5  (1,732) |  | 54.2  (7,231) | 48.9  (7,225) | 58.7  (5,342) | 59.1  (3,895) |
| Tanzania (2015-16) | 31.9  (8,123) | 34.8  (7,444) | 35.4  (7,256) | 36.2  (6,621) |  | 28.4  (3,081) | 20.9  (4,199) | 41.2  (1,532) | 46.0  (797) |  | 30.9  (11,204) | 29.8  (11,643) | 36.5  (8,788) | 37.3  (7,418) |
| Uganda (2016) | 34.7  (11,105) | 36.8  (10,187) | 37.3  (9,727) | 38.8  (8,463) |  | 24.9  (4,358) | 17.4  (5,618) | 41.6  (1,885) | 47.4  (861) |  | 31.8  (15,463) | 29.7  (15,805) | 38.0  (11,612) | 39.6  (9,324) |
| Zambia (2013-14) | 44.0  (8,730) | 47.2  (8,968) | 47.7  (8,761) | 48.7  (8,084) |  | 20.7  (4,381) | 14.8  (5,073) | 33.7  (1,802) | 37.5  (886) |  | 36.6  (13,111) | 35.8  (14,041) | 45.5  (10,563) | 47.6  (8,970) |
| Zimbabwe (2015) | 65.8  (5,971) | 67.4  (5,768) | 68.2  (5,557) | 69.6  (4,941) |  | 36.8  (2,104) | 19.7  (3,266) | 60.4  (859) | 66.6  (395) |  | 58.7  (8,075) | 50.9  (9,034) | 67.3  (6,416) | 69.4  (5,336) |
| **West and Central Africa** | | | |  |  |  |  |  |  |  |  |  |  |  |
| Benin (2011-12) | 8.2  (10,265) | 9.3  (8,316) | 10.2  (7,125) | 11.0  (5,840) |  | 16.7  (2,666) | 12.7  (3,831) | 23.8  (1,478) | 24.7  (869) |  | 10.0  (12,931) | 10.4  (12,147) | 12.6  (8,603) | 12.8  (6,709) |
| Chad (2014-15) | 4.7  (12,128) | 5.0  (12,037) | 5.0  (11,258) | 4.9  (10,034) |  | 8.9  (1,786) | 4.5  (3,385) | 19.5  (521) | 19.6  (313) |  | 5.3  (13,914) | 4.9  (15,422) | 5.7  (11,779) | 5.4  (10,347) |
| Democratic Republic of Congo (2013-14) | 7.8  (11,937) | 8.8  (10,555) | 8.8  (10,092) | 9.1  (8,972) |  | 13.4  (4,083) | 10.4  (5,034) | 21.2  (2,038) | 21.1  (1,267) |  | 9.3  (16,020) | 9.3  (15,589) | 11.0  (12,130) | 10.6  (10,239) |
| Côte d’Ivoire (2011-12) | 12.3  (5,976) | 14.3  (5,347) | 14.7  (5,040) | 15.3  (4,261) |  | 21.1  (2,672) | 18.5  (3,041) | 28.9  (1,741) | 30.3  (1,119) |  | 15.2  (8,648) | 15.9  (8,388) | 18.5  (6,781) | 18.6  (5,380) |
| Gabon (2012) | 20.0  (4,418) | 21.0  (4,119) | 21.6  (3,906) | 22.4  (3,281) |  | 35.8  (2,893) | 32.3  (3,059) | 44.1  (1,931) | 45.3  (1,237) |  | 26.8  (7,311) | 26.2  (7,178) | 29.8  (5,837) | 29.6  (4,518) |
| Gambia (2013) | 7.9  (6,408) | 9.6  (5,400) | 10.2  (4,847) | 10.4  (4,100) |  | 13.4  (732) | 3.5  (2,903) | 42.7  (169) | 42.0  (73) |  | 8.5  (7,140) | 7.4  (8,303) | 11.4  (5,016) | 11.0  (4,173) |
| Ghana (2014) | 21.9  (5,186) | 25.2  (4,520) | 26.1  (4,193) | 27.5  (3,292) |  | 18.2  (2,631) | 14.8  (3,209) | 30.0  (1,262) | 31.7  (653) |  | 20.6  (7,817) | 20.8  (7,729) | 27.0  (5,455) | 28.3  (3,945) |
| Guinea (2012) | 4.5  (6,552) | 4.7  (5,302) | 5.2  (4,121) | 5.2  (3,364) |  | 25.0  (1,252) | 14.9  (2,058) | 39.2  (675) | 41.1  (363) |  | 7.9  (7,804) | 7.6  (7,360) | 10.2  (4,796) | 8.8  (3,727) |
| Liberia (2013) | 19.4  (5,545) | 22.0  (4,782) | 22.6  (4,567) | 23.0  (3,872) |  | 26.7  (2,742) | 25.5  (2,685 | 33.8  (1,813) | 34.7  (1,237) |  | 22.2  (8,287) | 23.4  (7,467) | 26.4  (6,380) | 26.4  (5,109) |
| Mali (2012-13) | 9.8  (7,577) | 11.0  (7,056) | 11.1  (6,502) | 11.1  (5,881) |  | 17.9  (682) | 8.1  (1,438 | 28.2  (347) | 33.5  (196) |  | 10.4  (8,259) | 10.5  (8,494) | 11.9  (6,849) | 11.8  (6,077) |
| Niger (2012) | 12.4  (9,024) | 12.7  (8,726) | 13.6  (7,939) | 14.6  (6,926) |  | 4.6  (498) | 1.7  (1,326 | 28.1  (67) | 40.3  (25) |  | 12.0  (9,522) | 11.6  (10,052) | 13.7  (8,006) | 14.7  (6,951) |
| Nigeria (2013) | 9.7  (25,376) | 10.6  (24,231) | 10.8  (23,188) | 10.7  (20,706) |  | 29.9  (5,699) | 16.6  (9,994 | 50.3  (2,996) | 55.1  (1,783) |  | 13.1  (31,075) | 12.2  (34,225) | 14.8  (26,184) | 13.7  (22,489) |
| Sierra Leone (2013) | 15.7  (9,494) | 19.5  (7,833) | 20.7  (7,155) | 21.0  (6,264) |  | 42.0  (4,187) | 34.0  (5,185 | 52.5  (3,206) | 56.6  (2,117) |  | 23.6  (13,681) | 25.1  (13,018) | 30.2  (10,361) | 29.6  (8,381) |
| Togo (2013-14) | 17.4  (5,947) | 19.5  (5,301) | 20.5  (4,884) | 21.8  (3,849) |  | 24.4  (1,963) | 17.9  (2,606 | 36.6  (1,062) | 38.3  (600) |  | 19.2  (7,910) | 19.0  (7,907) | 23.4  (5,946) | 24.0  (4,449) |
| **West Asia/Europe** | | | |  |  |  |  |  |  |  |  |  |  |  |
| Albania (2017-18) | 3.6  (7,474) | 3.8  (7,018) | 3.9  (6,779) | 4.0  (6,047) |  | 3.8  (684) | 1.2  (3,005 | 8.0  (268) | 7.8  (193) |  | 3.6  (8,158) | 3.0  (10,023) | 4.1  (7,047) | 4.2  (6,240) |
| Armenia (2015-16) | 28.0  (3,998) | 28.3  (3,888) | 29.2  (3,756) | 29.9  (3,577) |  | 4.7  (379) | 0.9  (1,795 | 69.4  (20) | 87.3  (11) |  | 25.8  (4,377) | 19.2  (5,683) | 29.4  (3,776) | 30.0  (3,588) |
| Kyrgyz Republic (2012) | 33.5  (5,258) | 34.9  (5,165) | 35.9  (4,964) | 37.1  (4,674) |  | 11.5  (663) | 2.8  (2,312 | 31.9  (126) | 41.8  (59) |  | 30.6  (5,921) | 24.1  (7,477) | 35.8  (5,090) | 37.2  (4,733) |
| Tajikistan (2017) | 27.1  (7,499) | 28.7  (6,288) | 31.4  (5,388) | 33.1  (4,718) |  | 2.1  (710) | 0.3  (2,573 | 18.4  (31) | 24.5  (12) |  | 25.2  (8,209) | 20.9  (8,861) | 31.3  (5,419) | 33.0  (4,730) |
| **Asia and Pacific** | | |  |  |  |  |  |  |  |  |  |  |  |  |
| Cambodia (2014) | 38.6  (11,246) | 40.3  (11,028) | 41.0  (10,725) | 42.6  (9,651) |  | 4.7  (1,266) | 0.7  (4,918 | 31.6  (102) | 34.7  (50) |  | 35.2  (12,512) | 28.6  (15,946) | 41.0  (10,827) | 42.5  (9,701) |
| India (2015-16) | 47.7  (473,658) | 50.6  (95,767) | 52.3  (67,641) | 53.8  (56,771) |  | 32.9  (20,273) | 2.8  (180,715 | 36.0  (578) | 39.5  (247) |  | 47.1  (493,931) | 20.2  (276,482) | 52.2  (68,219) | 53.7  (57,018) |
| Indonesia (2012) | 57.8  (30,769) | 58.9  (30,477) | 60.2  (29,397) | 61.9  (25,730) |  | 5.3  (2,241) | 0.4  (11,203) | 12.7  (185) | 18.8  (71) |  | 54.4  (33,010) | 44.2  (41,680) | 60.0  (29,582) | 61.9  (25,801) |
| Myanmar (2015-16) | 51.4  (7,639) | 53.6  (7,139) | 55.8  (6,727) | 58.6  (5,804) |  | 2.7  (854) | 0.4  (4,344) | 20.8  (53) | 40.6  (19) |  | 46.6  (8,493) | 33.2  (11,483) | 55.5  (6,780) | 58.6  (5,823) |
| Nepal (2016) | 42.7  (9,860) | 47.2  (8,679) | 52.8  (7,322) | 56.3  (6,085) |  | 13.7  (347) | 0.3  (2,679) | 25.8  (16) | 78.3  (4) |  | 41.8  (10,207) | 36.0  (11,358) | 52.7  (7,338) | 56.3  (6,089) |
| Philippines (2017) | 40.4  (15,159) | 41.3  (13,609) | 42.7  (12,763) | 44.7  (10,701) |  | 7.5  (2,247) | 1.3  (8,249) | 16.4  (453) | 17.5  (224) |  | 35.7  (17,406) | 25.7  (21,858) | 41.7  (13,216) | 44.0  (10,925) |
| Timor Leste (2016) | 24.0  (7,424) | 22.6  (4,746) | 23.3  (4,433) | 25.3  (3,653) |  | 3.3  (458) | 0.0  (4,668) | 0.0  (84) | 0.0  (35) |  | 22.8  (7,882) | 11.5  (9,414) | 22.9  (4,517) | 25.0  (3,688) |
| **Latin America and Caribbean** | | | |  |  |  |  |  |  |  |  |  |  |  |
| Colombia (2015) | 75.9  (19,753) | 76.6  (19,142) | 77.2  (18,719) | 78.3  (16,918) |  | 58.2  (12,387) | 46.8  (13,940) | 71.6  (7,597) | 77.7  (4,594) |  | 68.7  (32,140) | 63.4  (33,082) | 75.4  (26,316) | 78.2  (21,512) |
| Dominican Republic (2013) | 68.2  (5,054) | 69.0  (5,076) | 69.8  (4,900) | 70.4  (4,497) |  | 49.1  (2,774) | 32.8  (3,575) | 57.9  (1,580) | 63.7  (920) |  | 61.1  (7,828) | 53.4  (8,651) | 66.7  (6,480) | 69.1  (5,417) |
| Guatemala (2014-15) | 48.8  (14,840) | 52.1  (13,711) | 53.3  (13,190) | 54.9  (12,033) |  | 22.1  (4,403) | 8.7  (8,586) | 44.7  (1,288) | 56.6  (697) |  | 42.6  (19,243) | 35.4  (22,297) | 52.5  (14,478) | 55.0  (12,730) |
| Haiti (2016-17) | 31.8  (7,554) | 33.7  (6,892) | 35.4  (6,443) | 36.5  (5,402) |  | 18.8  (4,291) | 13.7  (5,821) | 29.0  (2,232) | 30.6  (1,142) |  | 26.9  (11,845) | 24.4  (12,713) | 33.7  (8,675) | 35.4  (6,544) |
